# Protective Effects on 60Co-γ Radiation Damage of Pine Cone Polyphenols from *Pinus koraiensis*-Loaded Chitosan Microspheres In Vivo

**DOI:** 10.3390/molecules23061392

**Published:** 2018-06-08

**Authors:** Sujuan Shao, Juanjuan Yi, Joe M. Regenstein, Cuilin Cheng, Hua Zhang, Haitian Zhao, Zhenyu Wang

**Affiliations:** 1 Department of Food Science and Engineering, School of Chemistry and Chemical Engineering, Harbin Institute of Technology, Nangang District, Harbin 150090, China; 13322035260@163.com (S.S.); ccuilin@hit.edu.cn (C.C.); zhhua@hit.edu.cn (H.Z.); zhaoht9999@163.com (H.Z.); 2 School of Life Sciences, Zhengzhou University, Zhengzhou 450000, Henan, China; 18846044290@163.com; 3 Department of Food Science, Cornell University, Ithaca, NY 14853-7201, USA; jmr9@cornell.edu

**Keywords:** chitosan microspheres, radioprotection, pine cones, polyphenols, *Pinus koraiensis*

## Abstract

A novel chitosan microsphere for encapsulating pine cone polyphenols (PP) from *P. koraiensis* was successfully prepared using an emulsion crosslinking technique. The characteristics of pine polyphenol-loaded microspheres (PPM) were determined using scanning electron microscopy (SEM) and a laser particle size detector. It was found that PPMs were spherical in shape with uniform particle size distribution patterns. The drug content and encapsulation rate of the microspheres were 7.47% and 73.6%, respectively, at a Ch/GA mass ratio of 0.7. The animal experiments showed that PPM had a stronger radiation protective effect than PP. PPM significantly increased the immune organ indices, the quantity of marrow DNA, the superoxide dismutase (SOD) activity, the splenocyte proliferation index, and the phagocytosis activity of monocytes. PPM also decreased the numbers of micronuclei in bone marrow cells and malondialdehyde (MDA) levels in plasma in mice exposed to ^60^Co γ-irradiation. In addition, gender differences in biological responses to exposure to radiation were observed.

## 1. Introduction

Polyphenolic compounds, as naturally derived antioxidants, are receiving greater attention. *Pinus* species are rich in polyphenols. The polyphenols are found in pine bark, pine leaves, and pine cones [1]; and pine polyphenols (PP) have specific biological effects: anti-tumor [2], anti-inflammatory [3], antibacterial [4], antidiabetic [5], and antioxidant [6,7] activity. In particular, PPs have significant antioxidant and free radical scavenging activity in vitro and a protective effect against radiation induced damages in mice [8]. The conventional extraction of plant polyphenols involves aqueous or ethanol extractions. During these processes, polyphenolic compounds containing hydroxyl groups are reactive and can be damaged due to oxygen, temperature, pH, light, moisture, or other inappropriate storage conditions [9]. Furthermore, the unpleasant taste of polyphenols often limits their intake by humans [10]. According to recent reports, some of these difficulties might be alleviated by using encapsulation of microspheres. As a drug delivery system, it was found that microspheres could improve the stability and utilization of embedded drugs, promote drug absorption and stable release, and also prevent denaturation and degradation of antioxidant compounds [11].

Chitosan (Ch), cationic (1-4)-2-amino-2-deoxy-d-glucan, is biodegradable and histocompatable and is widely used as a food packaging material. Ch has a protective effect and can maintain the release of drugs [12]. Furthermore, there are a large number of free amino groups on the surface of Ch that will cross-link with glutaraldehyde (GA) and other bifunctional reagents. Based on this, Ch can be used to make microspheres, microcapsules, and other stable delivery systems. Ch has already been used for the encapsulation of probiotics and prebiotics [13], aromatic compounds [14], enzymes [15], and antioxidants [16,17]. Combining the polymer protective, mucoadhesive, and antimicrobial properties of Ch microspheres with the antioxidant activity of polyphenols could be useful. This research tried to prepare a novel chitosan microsphere for encapsulating PP by a GA emulsion crosslinking technique, in order to provide a natural stable antioxidant. 

Ionizing radiation (IR) is one of the most common treatments for human cancers. However, it also damages normal cells and tissues, especially within the immune system [18]. Exposure to IR damages biological macromolecules such as proteins, lipids, and DNA via direct and indirect pathways. IR also triggers the radiolysis of water in the cellular system and induces the generation of extraordinarily high levels of reactive oxygen species (ROS) in msec, which induces immediate and widespread oxidative damage [19]. Natural antioxidants without toxicity or side effects can help prevent and reduce the level of oxidative damage caused by free radicals and IR, which some people believe are safer and more reliable than synthetic antioxidants. Plant polyphenols have been found to have significant radioprotective effects. In a previous study from this laboratory, polyphenols from *Pinus koraiensis* had a strong antioxidant activity with the ABTS·C radical [20] and could effectively prevent injuries induced by γ-radiation in mice [21]. In this study, in order to explore the effect of microspheres prepared by emulsion crosslinking technique on radiation protection, Ch microspheres prepared using an emulsion technique as a carrier for PP were compared to free PP in vivo using mice.

## 2. Results and Discussion

### 2.1. Preparation of PPM

**Figure 1:**
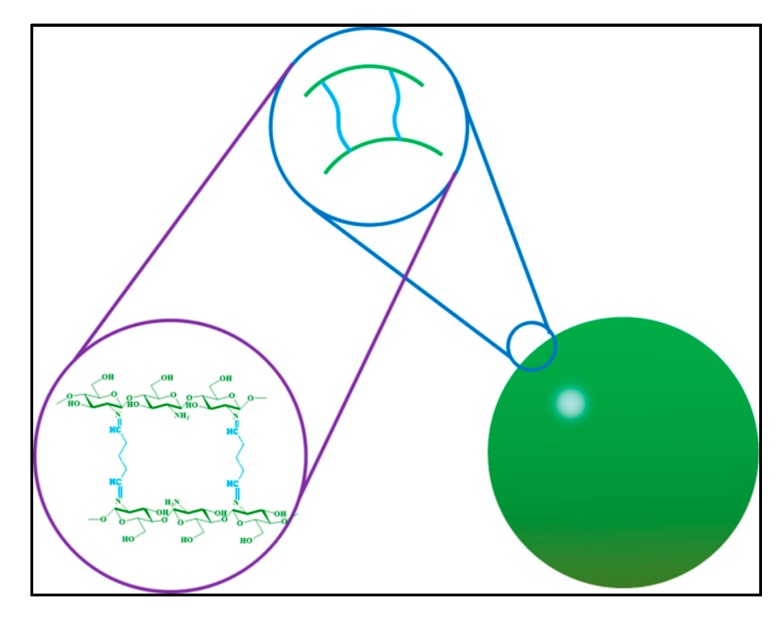
Emulsification cross-linking schematic.

Ch is a linear polyamine when at acidic pH, containing a number of free amine groups that are readily available for cross-linking; its cationic nature allows for ionic cross-linking with multivalent anions [22]. In this study, chemical cross-linking of chitosan microspheres was obtained using a well-known chemical cross-linking agent, glutaraldehyde (see Figure 1). It is known that the cross-linking mechanism involves formation of Schiff’s base structures [23]. In this method, a water-in-oil (w/o) emulsion is prepared by emulsifying the chitosan aqueous solution in the oil phase. Aqueous droplets are stabilized using a suitable surfactant. The stable emulsion is cross-linked using glutaraldehyde to harden the droplets. By this method, microspheres with a stable shape and uniform size can be prepared.

**Figure 2:**
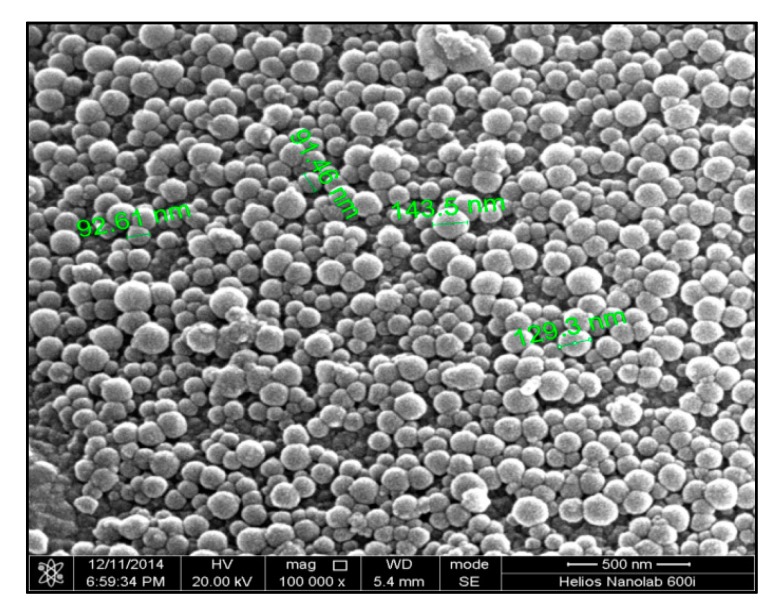
SEM picture of PPM.

**Figure 3:**
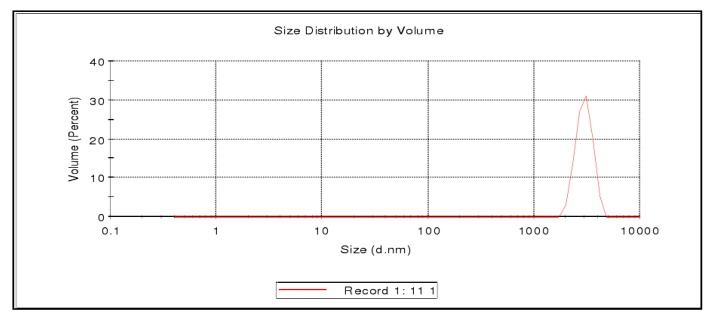
The size distribution of PPM.

The morphology of the resulting chitosan microspheres loaded with PP is shown in Figure 2. The image shows that all prepared microparticles were spherical with uniform size and had a smooth surface without any indentations or irregularities. The distribution curves of PP-loaded microspheres are shown in Figure 3. It could be observed that the vast majority of the particles studied had a diameter around 3.4 μm, which meant that the microspheres were successfully prepared.

According to a previous study from this laboratory, the degree of crosslinking and the surface morphology of the microspheres are evidently related to GA and Ch contents [24]. As shown in Table 1, the effect of different GA/Ch mass ratios on drug content and encapsulation efficiency of PPM was explored. The results revealed that PPM with the highest encapsulation rate and drug amount was at GA/Ch mass ratio of 0.7, calculated to be 73.57% and 7.47%, respectively. This might explain why, when the GA/Ch mass ratio is too low, the amino groups on the surface of chitosan are not completely involved in the reaction, making the GA and Ch unable to be crosslinked efficiently. However, when the GA/Ch mass ratio is too high, excessive cross-linking agent will break the oil–water system, leading to a reduction in the encapsulation efficiency and drug content of PPM.

### 2.2. Effect of PP and PPM on the Body Weight Changes of Mice

**Figure 4:**
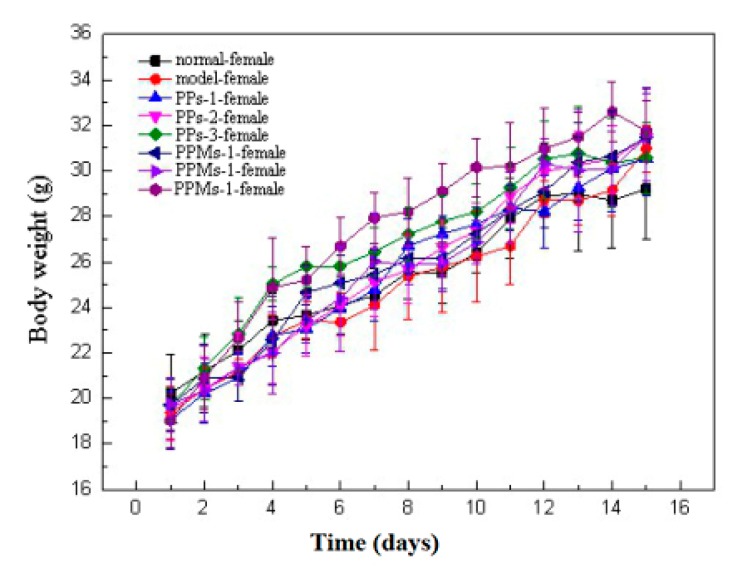
Effect of PP and PPM on the body weight changes of female mice.

**Figure 5:**
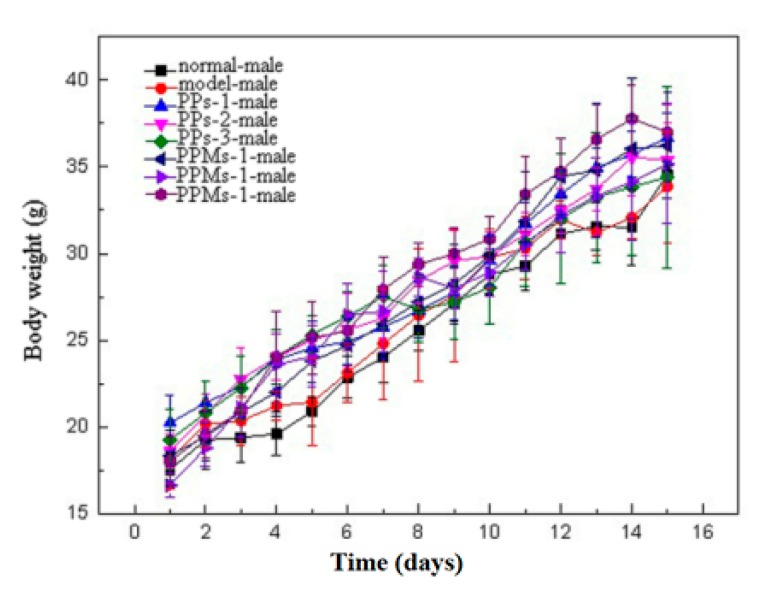
Effect of PP and PPM on the body weight changes of male mice.

The body weight changes of female and male mice over 15 days are shown in Figure 4 and Figure 5, respectively. The average body weight of mice increased slowly in the first three days, which was the process of mice adapting to the intragastric administration. Afterwards, the body weight of mice began to increase steadily. Compared with normal mice, the irradiated mice showed significant weight loss, accompanied by symptoms of different degrees of hair removal, lack of energy, decreased appetite, and low thirst. Compared with the model group, the mice in the PP and PPM groups showed a small decrease in mean body weight and relatively mild symptoms of radiation sickness. There was no significant difference in weight loss between PP and PPM in each dose group.

Whole-body radiation can damage the mouse digestive system, immune system, and hematopoietic system, causing weight loss in mice [21]. PP and PPM reduced the decline in body weight, which indicated they may play a radioprotective effect. When male and female mice in the model and treatment groups were compared, male mice gained more weight. (The level of significance is greater than 0.05, not shown in the figures.)

### 2.3. Effect of PP and PPM on the Thymus and Spleen Indices of Mice

The thymus and spleen are important immune organs that tend to shrink (lose weight) after bodies are injured by radiation. Therefore, indices of the thymus and spleen are commonly used to indicate the degree of radiation damage. Table 2 shows that the thymus and spleen indices of mice were significantly reduced after irradiation with 6 Gy ^60^Co γ-rays (*p* < 0.01), and all PP and PPM groups were much higher than that of the model group (*p* < 0.05), which indicates that PP and PPM had a protective effect on thymus and spleen injury induced by radiation. Compared with the PP group, the thymus and spleen indices of the PPM groups were mostly higher. This suggests that PPMs are more effective than PP. Furthermore, it was found that female mice in some groups were better able to maintain the weight of their immune organs after exposure to ionizing radiation, which indicated that there may be a gender difference in this indicator. These observations are similar to those of Reeve et al., which showed that male mice showed a relative unresponsiveness to the UVA-induced immune responses [25]. The authors speculated that the results were due to the estrogenic pathway in female mice being activated by the radiation, but more research is needed.

### 2.4. The Effect of PP and PPM on SOD Activity and MDA Levels

Ionizing radiation induced the cells to produce excessive ROS, which leads to lipid peroxidation, breaks redox homeostasis within cells and living tissues, generates MDA, and decreases the activities of enzymatic and non-enzymatic antioxidants in vivo [26,27,28]. In the present study, as shown in Table 3, administration of PP and PPM reduced the levels of MDA and restored the SOD activity of irradiated mice, which is consistent with a previous study that showed PP could reduce the redox imbalance and lipid peroxidation, and partly restore the redox balance of radiation-injured mice [21]. Many other studies also suggested that plant polyphenols had an anti-radiation and anti-oxidative stress effect by restoring the redox balance of the system [29,30,31]. The treatment with PPM was more effective than PP, which may be due to its higher bioavailability and more stable physical and chemical properties [24]. Another reason might be that Ch also had radioprotective effects [32,33]. When it was combined with PP, there may be a synergistic radiation protective effect.

In addition, it was found that after exposing mice to ionizing radiation, there were lower declines in serum SOD activity in female mice in the model groups than in male mice (*p* < 0.05). On the other hand, the female mice in the model groups produced less serum MDA than male mice (*p* < 0.01). This implies that there may be a gender effect in radiation-induced antioxidant defenses in mice. Future studies should determine whether female mice have more effective inducible endogenous antioxidant defenses compared to males.

### 2.5. Effect of PP and PPM on Bone Marrow Micronuclei Formation and Quantity of Marrow DNA

The main mechanism of radiation injury is induction of apoptosis or cell death through free radical-mediated DNA damage, including single or double-strand breaks and basic group damage, and the cross-linking of DNA molecules [34]. Ionizing radiation also acts directly on nucleic acids, proteins, and enzymes, causing ionization and breakage of chemical bonds, leading to destruction of DNA and cell damage [35]. The bone marrow is damaged at the molecular level by radiation, which causes chromosome aberrations and increased development of micronuclei [36]. Micronucleus detection and DNA content can, therefore, be used as a diagnostic biological indicator of radiation damage.

**Figure 6:**
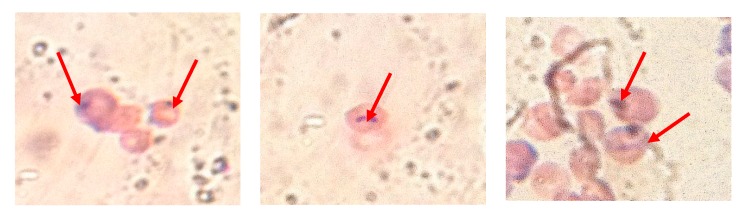
Bone marrow micronucleus pictures.

**Figure 7:**
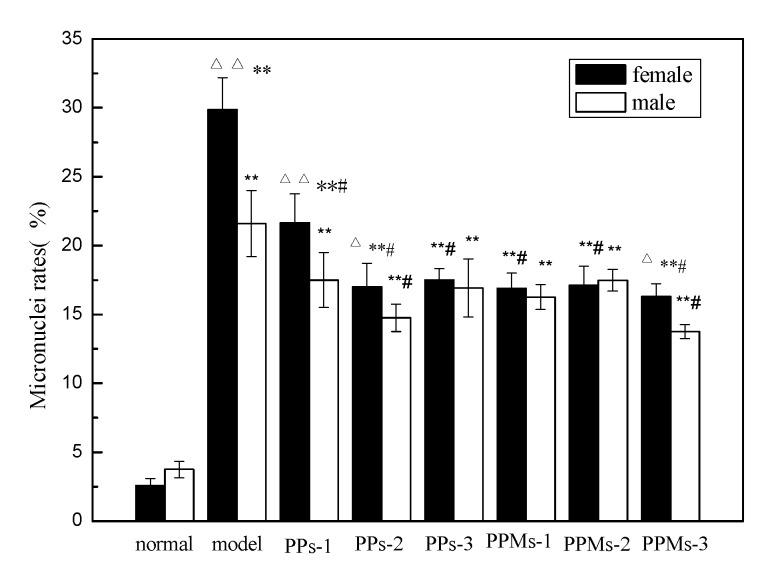
Effect of PP and PPM on the development of micronuclei in bone marrow; the irradiation dose was 6 Gy. * *p* < 0.05 and **p < 0.01 compared with the normal group; ^#^ *p* < 0.05 compared with the model group; ^Δ^ *p* < 0.05 compared with the male group in the same indicator and ^ΔΔ^ *p* < 0.05 compared with the male group in the same indicator (mean ± SD, *n* = 6).

Ionizing radiation disrupts normal cell division and differentiation, resulting in increased micronuclei in cells. The arrow in Figure 6 indicates common bone marrow micronucleus. The more micronuclei that appear, the more severely the bone marrow cells are damaged. Different groups of mice bone marrow micronuclei are shown in Figure 7. Compared with the model group, the bone marrow micronucleus rate of the treatment groups was significantly lower. These results indicated that PP and PPM inhibited the production of radiation-induced micronuclei and, therefore, had a protective role. This is consistent with the study of tea polyphenols against radiation [37].

**Figure 8:**
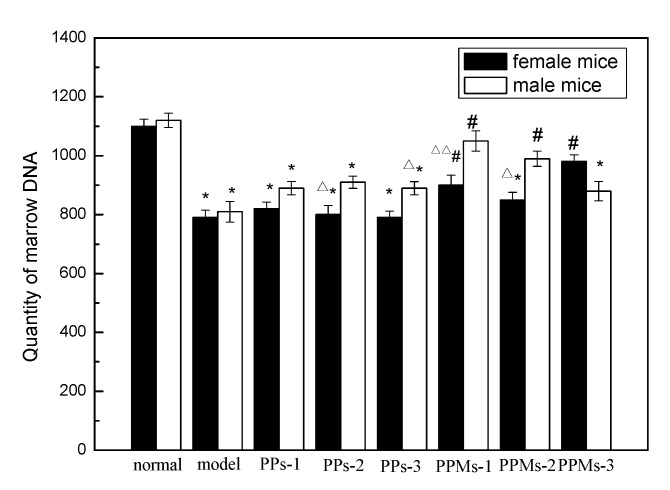
Effect of PP and PPM on quantity of marrow DNA; the irradiation dose was 6 Gy. * *p* < 0.05; ^#^ *p* < 0.05 compared with the model group; ^Δ^ *p* < 0.05 compared with the male group in the same indicator and ^ΔΔ^ *p* < 0.01 compared with the male group in the same indicator (mean ± SD, *n* = 6).

Figure 8 shows the effect of PP and PPM on the quantity of marrow DNA in mice. Total body irradiation significantly reduced the quantity of bone marrow DNA (*p* < 0.05). The DNA contents of the bone marrow in the PP and PPM were generally higher than in the model group. However, the DNA content of bone marrow cells in some groups of PPM was not significantly different from those in the normal group (*p* > 0.05), which suggests that PP and PPM can reduce the radiation damage to bone marrow cells in mice, with the effects of PPM being stronger than PP. Previously, the radiation protection indicators of PP and PPM in the female mice groups were stronger than in the male mice groups, while the bone marrow micronucleus and DNA content were the opposite. Further work is needed to clarify this discrepancy.

### 2.6. Effect of PP and PPM on the Splenocyte Proliferation Index

**Figure 9:**
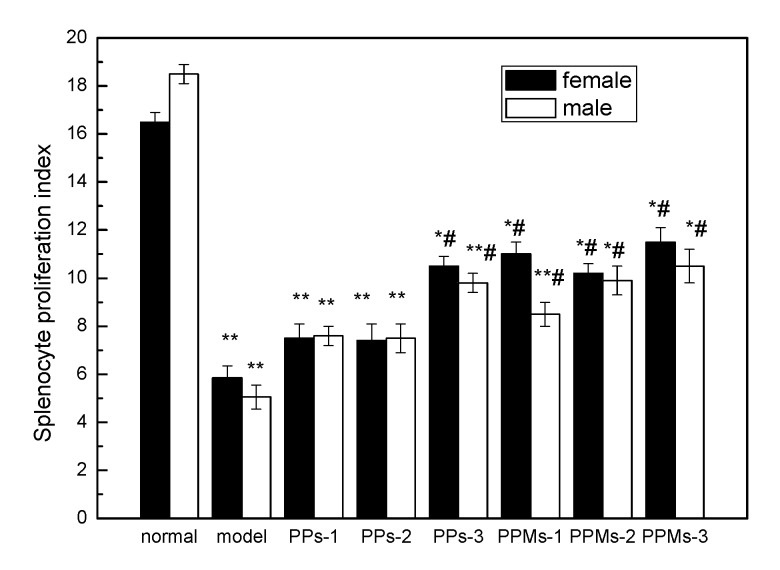
Effect of PP and PPM on the proliferation of splenic lymphocytes; the irradiation dose was 6 Gy. * *p* < 0.05 and ** *p* < 0.01 compared with the normal group; ^#^ *p* < 0.05 compared with the model group (mean ± SD, *n* = 6).

The immune system plays an important role in radiation protection. T-lymphocytes are some of the most important immunologically active cells and play a significant role in enhancing immune function. The results in Figure 9 showed that, even with the stimulation of Con A, the proliferation ability of lymphocytes in radiation injury of mice was significantly lower than that in normal mice (*p* < 0.05), which indicated that radiation caused damage to spleen lymphocytes of mice. PP and PPM reduced the damage and promoted the proliferation ability of Con A-stimulated splenocytes compared with the model control group. T-lymphocytes play a central role in the generation and regulation of the immune response to radiation and oxidative stress. PP and PPM significantly increased the activation of T-lymphocytes and enhanced humoral-mediated immune responses in irradiated mice. These results were similar to those of Yi [24]. Similarly, the PPM groups mostly did better than PP, while female mice were better than male mice, but no significance was found.

### 2.7. Effect of PP and PPM on Phagocytosis of Monocytes

**Figure 10:**
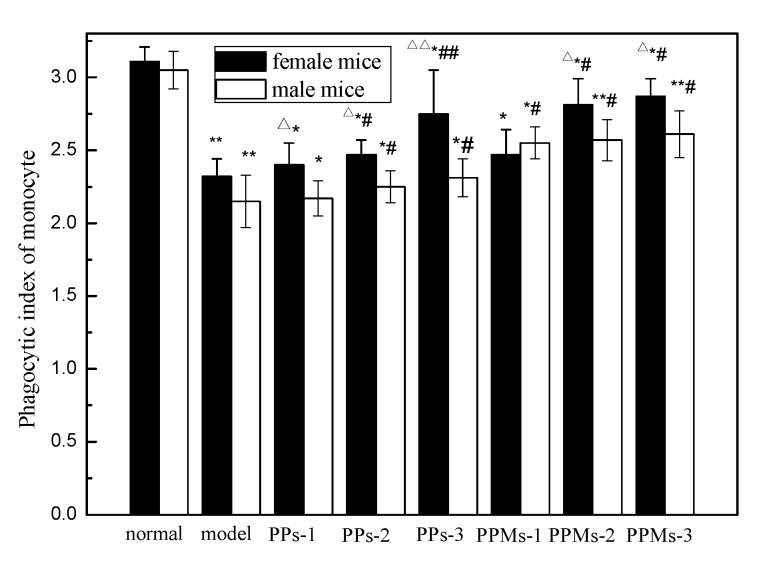
Effect of PP and PPM on phagocytosis of monocytes; the irradiation dose was 6 Gy. * *p* < 0.05 and ** *p* < 0.01 compared with the normal group; ^#^ *p* < 0.05 compared with the model group and ^##^ *p* < 0.01 compared with the model group; ^Δ^ *p* < 0.05 compared with the male group in the same indicator and ^ΔΔ^ *p* < 0.01 compared with the male group in the same indicator (mean ± SD, *n* = 6).

Monocytes are important immune phagocytes in the body. The phagocytosis of monocytes is often used as an indicator of PP’s protective role with immune cell function. Figure 10 shows the effect of PP and PPM on the phagocytosis in female and male mice. Compared with the normal group, the phagocytic index (PI) of the model group was significantly decreased (*p* < 0.05). After administration of PP and PPM, the PI of all treatment groups were increased. With the same polyphenol conditions, the phagocytic index of the PPM groups was higher than PP groups, with a dose-dependent trend. The result confirmed that PPM has strong monocyte phagocytosis activity in vivo. Similarly, gender differences in the PI were also observed between females and males. Macrophages in irradiated female mice were less damaged. The sex differences may be affected by different levels of sex hormones. It has been reported that both estrogen and testosterone may modify the organism’s response to irradiation. One study pointed out that such differences can occur pre-puberty [38].

## 3. Materials and Methods

### 3.1. Materials

The dried pine cones of *Pinus koraiensis* were provided by Yichun Hongxing District Forestry Bureau (Yichun, China). *N*-carboxymethyl Ch (with a degree of deacetylation of 92% according to the manufacturer) was purchased from Tianjin University Kewei Co. (Tianjin, China). The superoxide dismutase (SOD) and malondialdehyde (MDA) measurement kits were purchased form Nanjing Jiancheng Bioengineering Institute (Nanjing, China). All other chemicals were of analytical grade purchased from local suppliers. 

PP from *Pinus koraiensis* were prepared according to the method of Li & Wang [21] and enriched using an X-5 macroporous resin chromatographic column (Shengda Co., Ltd., Harbin, China). The purity of the collected polyphenols was 40.2% according to the Folin–Ciocalteau method. 

### 3.2. Preparation of PPM

Pine polyphenol-loaded microspheres (PPM) were prepared using the traditional method of emulsion cross-linking and slightly modified using the method of An [39]. The particles were prepared at a chitosan concentration of 2.0% (*w*/*v*), as well as at different GA/Chmass ratios: 0.5, 0.7, and 0.9. The aqueous phase of emulsion (solution of chitosan and 3% (*v*/*v*) of acetic acid in water) was mixed in a 1:12 ratio with an oil phase (paraffin oil with addition of span 80 (3.0% (*v*/*v*)). Prior to mixing, solutions were purged with nitrogen to remove oxygen. After homogenization, a certain amount of GA was added drop by drop; stirring was continued for 1 h using a magnetic stirrer (at a speed of 600 r/min), allowing the microparticles to harden. The obtained microparticles were washed three times with distilled water, ethanol (analytical purity), and petroleum ether (analytical purity), respectively, followed by continued rinsing with distilled water until complete removal of surfactant. Washed microparticles were dried in an oven (DZF-6050, Zhongjing Science Instrument Co., Ltd., Beijing, China) at 50 °C and then stored in a desiccator until further use.

### 3.3. Characterizations of PPM

PPMs were subjected to particle-size distribution analysis using a laser particle size detector (Malvern Instruments, Malvern, UK). The morphology was observed using an XL30-ESEM (Environmental Scanning Electron Microscope (SEM), Philips Co., Amsterdam, The Netherlands) at 10 kV with a magnification of 3000×. The samples were sprinkled on to conductive glue (Electrodag, 3M4490, Zhongjing Science Instrument Co., Ltd., Beijing, China) on a copper SEM stub and sputter coated with gold (sputter coater, SCD004, BALTEC Balzers, Fürstentum, Liechtenstein).

### 3.4. Determination of PP Content and Entrapment Efficiency

PPM suspensions were centrifuged at 15,000 r/min at 4 °C for 30 min. The free PP in the clear supernatant was determined in triplicate using the polyphenols colorimetric assay method of Fan et al. [40]. The PP content and entrapment efficiency of PPM were calculated using the following equations:

(1) PP content = (A−B)/C×100

(2) Encapsulation efficiency (%) = (A−B)/A×100,

where A is the total amount of pine cone polyphenols in the initial solution (mg); B is the total amount of pine cone polyphenols in the supernatant (mg); and C is the weight of the nanoparticles measured after freeze-drying (mg).

### 3.5. Animals

Male and female ICR (Institute of Cancer Research) mice, 4–6 weeks old, with body weights of ~20 ± 2 g were provided by the Harbin Medical University (Harbin, China). The mice were housed in a mouse room at room temperature (25 °C) with a 12-h light/dark cycle and were provided with free access to standard mouse chow and water ad libitum. The experimental protocols were approved by the Heilongjiang University of Chinese Medicine animal ethics committee (SCXK Hei 200,800,4). All efforts were made to minimize animal suffering.

### 3.6. Oral Administration and Irradiation

Mice were randomly divided into 16 groups of 20 animals each, half male and half female, and included a control group, a group that was irradiated without any pretreatment and groups treat with PP: PP-1 (25 mg/kg), PP-2 (50 mg/kg) and PP-3 (100 mg/kg) and groups treated with PPM with equivalent amounts of PP to the above: PPM-1, PPM-2, PPM-3. After two weeks of treatment, all of the animals except for the control groups received full body radiation. Then the animals were fasted overnight prior to being sacrificed.

The ^60^Co irradiator of the Heilongjiang Academy of Agricultural Sciences was used for the irradiation experiments. Unanesthetized mice were restrained in well-ventilated boxes and exposed to whole-body ^60^Co γ-radiation (6 Gy), at a dose rate of 1 Gy/min at a source-to-animal distance (midpoint) of 400 cm.

### 3.7. Body Weight Changes

The body weight (BW) of each group of mice was recorded daily until they were sacrificed.

### 3.8. Index of Thymus and Spleen

On the day after irradiation animals were sacrificed, and the spleen and thymus were removed. Their organ indices were calculated by dividing the organ weight by the body weight (BW) [26]:

(3) Thymus index (%) = Thymus weight (g)/BW (g)×100

(4) Spleen index (%) = Spleen weight (g)/BW (g)×100.

### 3.9. The Effect of PP and PPM on SOD Activity and MDA Levels

Eyeball blood was collected from the mice. The blood was centrifuged for 15 min at 3500 r/min at 4 °C to obtain plasma. Homogenates (10% *w*/*v*) were prepared in normal saline. SOD activity and MDA levels of plasma were determined using commercial kits (Nanjing Jiancheng Bioengineering Institute, Nanjing, China) according to the manufacturer’s instructions. The SOD activity was expressed as U/mg protein, and the content of MDA was expressed in nmol/mg protein. 

### 3.10. Bone Marrow Micronuclei Rates and Quantity of Marrow DNA

Breastbones were obtained after mice were sacrificed. They were washed with 5 mL sodium phosphate-buffered saline (PBS) and centrifuged at 1200 r/min for 10 min at 4 °C. The supernatant was discarded and the sediment was applied to a slide covered with serum. The smear was fixed in methanol solution (analytical purity) for 10 min and then stained with Giemsa stain for 10–15 min. The presence of micronuclei was observed in evenly-dispersed visual fields using a high-power (400 magnification) microscope (XSP-1C, Zhongjing Science Instrument Co., Ltd., Beijing, China). The micronuclei rates (%) were calculated as the number of micronuclei in 1000 polychromatic erythrocytes [41]. 

Bone marrow was flushed from the femurs using 10 mL CaCl_2_ (5 mmol/L) and collected in centrifuge tubes at 4 °C for 30 min. Samples were centrifuged at 3000 r/min for 10 min at 4 °C. The supernatant was discarded. HClO_4_ (5 mL, 0.2 mol/L) was added to the precipitates and heated at 90 °C for 15 min. After cooling, they were centrifuged at 4000 r/min for 10 min, to obtain the supernatant. The absorbance value was determined at 268 nm [41].

(5) DNA content (μg) =40×50×A (absorbance)

### 3.11. Splenocyte Proliferation Index

The spleens, after weighing, were ground into small pieces and passed through a sterilized mesh (200 mesh) (Shengda Co., Ltd., Harbin, China) to obtain a cell suspension at room temperature. The red blood cells were removed using a 30% hemolytic red blood cell lysis solution (Solarbio, Beijing, China). Recovered splenocytes were washed twice, then re-suspended in RMPI-1640 complete medium, with 5 × 10^6^ cell/mL cell concentration [42]. The cells were seeded in a 96-well plate with or without Concanavalin A, an agent that promotes cell division (Con A, 7.5 μg/mL). After incubation for 72 h at 37 °C in a humidified 5% CO_2_ incubator, the number of cells was determined using MTT assay with a microplate reader (Model 680, Bio-Rad, Hercules, CA, USA) [43]. 

### 3.12. Phagocytosis of Monocytes

The phagocytosis function of monocytes was determined using the method of Yi [44]. Twenty-four hours after mice were irradiated, 25% (*v*/*v*) India ink was injected into the tail intravenously at 100 mL/kg body weight. A total of 20 μL of blood was collected through the eye orbit after 2 min (t1) and 10 min (t2), and added to 2 mL of 0.1% Na_2_CO_3_. The absorbance of blood at 600 nm after 2 min (A1) and 10 min (A2) was measured, and the absorbance of the control group was set to zero. The mice were sacrificed by decapitation, and then the liver and spleen were weighed. The clearance index (K) and phagocytic index (α) were calculated as follows [44]:

(6) K = (log A1− log A2)/(t1 − t2)

(7) $$\alpha \;={\;K}^{1/3}\times \;\mathrm{body}\;\mathrm{weight}/\left(\mathrm{liver}\;\mathrm{weight}\;+\;\mathrm{spleen}\;\mathrm{weight}\right).$$

### 3.13. Statistical Analysis

All statistical analyses used SPSS for Windows, Version 18.0. Data were expressed as means ± standard deviation (SD) of three independent measurements. Statistical analyses were done using one-way ANOVA. Differences at *p* < 0.05 and *p* < 0.01 were considered statistically significant using Duncan’s new multiple range test.

## 4. Conclusions

In this study pine polyphenol-loaded microspheres were successfully prepared. Animal experiments were used to measure a series of biological indicators, which showed that PPMs have a protective effect on ^60^Co γ-radiation induced damages in mice, and the effect is stronger than PP itself. This may be due to its high bioavailability, stable properties, and Ch’s anti-radiation effects. Female mice generally have higher resistance to radiation than male mice, although the mechanism remains to be further studied.

## Figures and Tables

**Table 1:** The drug content and encapsulation efficiency of PPM.

Sample	GA/Ch Mass Ratio	Drug Content (%)	Encapsulation Rate (%)
I	0.5	68.49	5.51
II	0.7	73.57	7.47
III	0.9	70.14	6.43

**Table 2:** Thymus and spleen indices.

Group	Thymus—Female (%)	Thymus—Male (%)	Spleen—Female (%)	Spleen—Male (%)
Normal	0.17 ± 0.87	0.18 ± 0.01	0.18 ± 0.08	0.18 ± 0.08
Model	0.11 ± 0.03 **	0.10 ± 0.09 **	0.14 ± 0.06 **	0.14 ± 0.01 **
PP-1	0.15 ± 0.01 ^#^	0.14 ± 0.01 *^,#^	0.17 ± 0.03 ^Δ^^,^*^,^^#^	0.15 ± 0.07 *^,#^
PP-2	0.15 ± 0.01 *^#^	0.15 ± 0.03 *^,#^	0.16 ± 0.12 *^,#^	0.17 ± 0.01 *^,#^
PP-3	0.16 ± 0.07 ^Δ^^,##^	0.14 ± 0.05 **^,#^	0.15 ± 0.34 **	0.16 ± 0.01 *^,#^
PPM-1	0.14 ± 0.04 ^ΔΔ^^,^**^,#^	0.18 ± 0.08 ^##^	0.16 ± 0.09 *^,#^	0.17 ± 0.09 ^##^
PPM-2	0.19 ± 0.07 ^Δ^^,^*^,##^	0.16 ± 0.06 *^,#^	0.19 ± 0.05 ^Δ^^,^*^,#^	0.16 ± 0.03 *^,#^
PPM-3	0.17 ± 0.11 ^##^	0.16 ± 0.03 *^#^	0.19 ± 0.08 ^ΔΔ^^,#^	0.15 ± 0.04 *^,#^

The irradiation dose was 6 Gy. * *p* < 0.05 and ** *p* < 0.01 compared with the normal group; ^#^ *p* < 0.05 compared with the model group and ^##^ *p* < 0.01 compared with the model group; ^Δ^ *p* < 0.05 compared with the male group in the same indicator and ^ΔΔ^ *p* < 0.05 compared with the male group in the same indicator (mean ± SD, *n* = 6).

**Table 3:** SOD activity and MDA levels in serum of different groups of mice.

Group	SOD-Female (U/L)	SOD-Male (U/L)	MDA-Female (nmol/mL)	MDA-Male (nmol/mL)
Normal	105 ± 5 ^Δ^	102 ± 3	1.6 ± 0.5	1.7 ± 0.8
Model	85 ± 2 ^Δ^^,^*	67 ± 7 **	4.8 ± 0.3 ^ΔΔ^^,^**	6.7 ± 1.0 **
PP-1	75 ± 3 ^Δ^^,^**^,#^	84 ± 5 *^,#^	3.2 ± 0.4 ^Δ^^,^**^,#^	5.7 ± 0.8 **
PP-2	82 ± 5 ^Δ^^,^*	73 ± 3 *^,#^	3.7 ± 0.6 ^Δ^^,^**^,#^	4.6 ± 0.4 **^,#^
PP-3	87 ± 2 ^Δ^^,^*	79 ± 3 *^,#^	4.0 ± 0.2 ^Δ^^,^**	2.7 ± 0.5 ^#^
PPM-1	80 ± 4 ^Δ^^,^**^,#^	74 ± 3 *	2.9 ± 0.4 *^,#^	3.4 ± 0.1 *^,#^
PPM-2	89 ± 3 *	89 ± 4 *^,#^	3.1 ± 0.9 ^Δ^^,^*^,#^	4.6 ± 0.7 **^,#^
PPM-3	90 ± 3 *^,#^	87 ± 2 *^,#^	3.2 ± 0.2 **^,#^	3.2 ± 0.7 *^,##^

The irradiation dose was 6 Gy. * *p* < 0.05 and ** *p* < 0.01 compared with the normal group; ^#^ *p* < 0.05 compared with the model group and ^##^ *p* < 0.01 compared with the model group; ^Δ^ *p* < 0.05 compared with the male group in the same indicator and ^ΔΔ^ *p* < 0.05 compared with the male group in the same indicator (mean ± SD, *n* = 6).
